# The prevalence of sleep disturbance among asthmatic patients in a tertiary care center

**DOI:** 10.1038/s41598-020-79697-x

**Published:** 2021-01-28

**Authors:** Tammam M. Alanazi, Hazim S. Alghamdi, Meshal S. Alberreet, Abdulaziz M. Alkewaibeen, Abdulrahman M. Alkhalefah, Aamir Omair, Hamdan AL-Jahdali, Abdullah AL-Harbi

**Affiliations:** 1 College of Medicine, King Saud Bin Abdulaziz University for Health Sciences (KSAU-HS), Riyadh, Saudi Arabia; 2 Pulmonary Division, Department of Medicine, Ministry of National Guard-Health Affairs, King Abdulaziz Medical City, MC 1443, P.O. Box 22490, Riyadh, 11426 Saudi Arabia; 3 King Abdullah International Medical Research Center, Riyadh, Saudi Arabia; 4 Department of Medical Education, College of Medicine, King Saud Bin Abdulaziz University for Health Sciences, Riyadh, Saudi Arabia

**Keywords:** Risk factors, Signs and symptoms, Respiratory tract diseases, Asthma, Sleep disorders

## Abstract

Sleep disturbances are commonly reported by patients with asthma. However, the prevalence of sleep disturbance and its association with the level of asthma control is unknown. The primary objective was to determine the prevalence of sleep disturbance among Saudi adult asthmatic patients attending pulmonary clinics at King Abdulaziz Medical City (KAMC). The study also aimed to compare sleep quality between controlled and uncontrolled asthma patients. The study was carried out in the outpatient pulmonary clinics at KAMC and utilized a cross-sectional survey. The survey included five different questionnaires: asthma control test and questionnaires related to the quality of sleep (Pittsburgh sleep quality index [PSQI], Epworth sleepiness scale [ESS], Berlin questionnaire [a measure of obstructive sleep apnea risk], and insomnia severity index [ISI]). Among the 200 asthma patients, 66% suffered from poor sleep quality (PSQI > 5), 43% were at high risk for obstructive sleep apnea, 25% had excessive daytime sleepiness (ESS > 10), and 46.5% had significant clinical insomnia (ISI ≥ 10). Poor sleep quality was less common in patients with well-controlled asthma (37%) compared to those with partially controlled asthma (78%) and uncontrolled asthma (82%) (p < 0.001). Poor sleep quality was common among patients with asthma, particularly those with suboptimal levels of asthma control. Further studies are needed to better understand the interaction between these two conditions.

## Introduction

Asthma is a common chronic disease characterized by recurrent symptoms, variable airflow obstruction, bronchial hyper-responsiveness (BHR), and underlying inflammatory changes affecting mainly the airways^1,2^. It is estimated approximately 300 million people suffering from asthma worldwide^2^. A recent meta-analysis estimated pooled weighted prevalence rate of asthma is14.3% among Saudi population^3^.

Sleep disorders are characterized by the inability to maintain good quality sleep or problems with the duration of sleep, which can have an impact on daytime activities. Examples of sleep disorders include obstructive sleep apnea (OSA), insomnia, difficulty maintaining sleep, and restless leg syndrome. Sleep disturbances are common complaints among asthma patients and can be manifested as difficulties initiating and maintaining sleep, as well as early morning awakenings^4–7^. Sleep disturbance at night may be partly due to poor asthma control and can also be related to diurnal variations in airflow limitation, with an increase in BHR and airway resistance at night^8–10^.

Other common conditions, such as gastroesophageal reflux disease (GERD), OSA, chronic rhinitis, and depression, are associated with asthma and can also lead to sleep disturbances and poor sleep quality independent of asthma control^8,11–13^. In addition, asthma is associated with a higher prevalence of OSA, especially in patients with difficult-to-control asthma^14–16^. A recent meta-analysis showed that the prevalence of OSA in adult asthma patients is estimated to be 50%, with an odds ratio of 2.64 for OSA in asthma patients compared with non-asthma patients^15^. Poor sleep quality was associated with worse asthma control and quality of life, even after controlling for GERD and other covariates^16^.

Insomnia is also common among asthma patients. Thirty-seven percent of asthma patients have clinically significant insomnia, which has been associated with worse asthma symptoms, poor quality of life, and increased asthma-related health care utilization in the preceding year^17^. Furthermore, daytime sleepiness from sleep disturbance has been shown to affect 30–50% of asthma patients^5,18^.

The association between asthma severity and sleep disturbance is quite complex and needs to be addressed. The purpose of this study was to determine the prevalence of sleep disturbance among a sample of Saudi adult asthmatic patients. We also aimed to compare sleep quality between controlled and uncontrolled asthma patients.

## Materials and methods

This cross-sectional study was conducted in the outpatient general pulmonary clinics at King Abdulaziz Medical City (KAMC) in Riyadh, Saudi Arabia. The study was approved by the Institutional Review Board Committee at King Abdullah International Medical Research Center. All patients who had a diagnosis of asthma and were scheduled to visit the pulmonary clinics from August 2018 to April 2019 were screened for eligibility. The inclusion criteria were the following; (1) ages 15 years and older; and (2) confirmed cases of bronchial asthma based on previous documentation of reversible obstructive airway disease with a positive response to a bronchodilator. Current smokers and patients with a history of > 10 pack years of smoking were excluded from the study. We also excluded patients with OSA, obesity hypoventilation syndrome, chronic obstructive pulmonary disease, other chronic lung diseases, heart failure, neuromuscular diseases, or other severe chronic systemic illnesses that may affect sleep quality.

Patients were approached by a co-investigator, who explained the purpose of the study. Written informed consent was then obtained from individual or guardian participants, and from the parent for patient who are less than 16 years old. Patient was asked to complete the questionnaires. The questionnaires included seven sections: demographics, Asthma Control Test (ACT), common sleep disorders, risk of OSA (Berlin questionnaire [BQ]), excessive sleepiness (Epworth Sleepiness Scale [EES]), sleep quality (Pittsburgh Sleep Quality Index [PSQI], and insomnia (Insomnia Severity Index [ISI]). All of these questionnaires have been previously validated in Arabic^22,24,26,28,30^.

The ACT evaluates the level of asthma control and ranges from 5 (poor control) to 25 (complete control), with higher scores reflecting greater asthma control. An ACT score > 19 indicates well-controlled asthma^19–22^. The PSQI assesses sleep quality during the past month and contains seven component scales: sleep quality, sleep latency, sleep duration, sleep efficiency, sleep disturbances, use of sleep medication, and daytime dysfunction. Each component is scored from 0 to 3, yielding a global PSQI score ranging from 0 to 21, with higher scores indicating worse sleep quality. A global PSQI score > 5 has a sensitivity of 89.6% and specificity of 86.5% in differentiating good from poor sleepers^23,24^.The ESS measures daytime sleepiness and rates the likelihood of dozing in eight specific situations, with scores from 0 (no chance of dozing) to 3 (high chance of dozing). Total scores range from 0 to 24, and a score > 10 on the ESS is indicative of excessive daytime sleepiness^25,26^. The BQ identifies patients with a high risk for OSA by assessing snoring severity, excessive daytime sleepiness, history of high blood pressure, and history of obesity. The overall risk score (high/low risk) was used in the analysis^27,28^. Lastly, the ISI measures the nature, severity, and impact of insomnia. It assesses severity of sleep onset, sleep maintenance and early morning wakening problems, sleep dissatisfaction, interference of sleep difficulties with daytime functioning, noticeability of sleep problems by others, and distress caused by the sleep difficulties. Total scores range from 0 to 28, and a cutoff score of ≥ 10 has an 86.1% sensitivity and 87.7% specificity for detecting insomnia cases^29,30^.

### Statistical analysis

All analyses were conducted using Statistical Package for Social Sciences (SPSS; v. 25.0). Continuous variables are summarized as means ± standard deviations (SD) or medians and interquartile range (IQR) for normal and non-normal distributions, respectively. Categorical data are described by frequencies and percentages. The one-way analysis of variance test was used to compare the means of continuous variables, and the chi-square test was used to compare the proportion of sleep disturbance between patients with different levels of asthma control. Potential risk factors causing poor sleep quality in asthma patients, including age, body mass index (BMI), gender, GERD, and risk of sleep apnea, were analyzed using univariate and multivariate analyses. To identify factors that were strongly associated with poor sleep quality, logistic regression analysis was used. A p value < 0.05 was considered statistically significant.

### Ethics approval and consent to participate

The study was approved by the institutional review board of King Abdullah International Medical Research Center (KAIMRC) in Riyadh, Saudi Arabia. All procedures performed in studies involving human participants were in accordance with the ethical standards of the institutional and/or national research committee and with the 1964 Helsinki declaration and its later amendments .Written informed consent was obtained from individual or guardian participants, and from the parent for patient who are less than 16 years old.

## Results

The study was conducted from August 2018 to April 2019. A total of 200 asthma patients participated in the study and filled out the questionnaires, with a response rate of 90%. Baseline patient characteristics included a mean age of 50.6 ± 17 years and female predominance (67%). According to the ACT, 67 (33.5%) patients had well-controlled asthma, 55 (27.5%) had partially controlled asthma, and 78 (39%) had uncontrolled asthma. Comorbid conditions were present in almost half of the patients (Table 1).

**Table 1:** Baseline characteristics in patients with different levels of asthma control.

	All (N = 200)	Asthma control levels			p value
		Well-controlled (N = 67)	Partially controlled (N = 55)	Uncontrolled (N = 78)	
Age (years)	50.6 ± 17.1	48.7 ± 18.7	52.3 ± 16.6	51 ± 16.1	0.49
Female gender	135 (67%)	41 (61%)	37 (67%)	57 (73%)	0.31
BMI (kg/m^2^)^a^	31.2 (7.5)	28.9 (7.4)	32.7 (6.8)	32.1 (7.4)	0.008
Never smokers	193 (96%)	65 (97%)	52 (94%)	76 (97%)	0.65
Coffee/tea intake	4 (2, 6)	4 (2, 6)	4 (2, 7)	4 (2, 7)	0.89
Educated	176 (88%)	59 (88%)	50 (91%)	67 (86%)	0.68
Married	146 (73%)	47 (70%)	44 (80%)	55 (71%)	0.39
**Co-morbidities**					
+ Allergic rhinitis	96 (48%)	35 (52%)	25 (45%)	36 (46%)	0.69
+ GERD	38 (19%)	15 (22%)	12 (22%)	11 (14%)	0.37
+ Hypertension	90 (45%)	26 (39%)	29 (53%)	35 (45%)	0.31
+ Diabetes mellitus	71 (36%)	24 (36%)	21 (38%)	26 (33%)	0.85
**Sleep duration**					
Less than 6 h	95 (47%)	23 (34%)	33 (60%)	39 (50%)	0.02
More than 6 h	105 (53%)	44 (66%)	22 (40%)	39 (50%)	

All other results are reported as means ± standard deviations or frequencies (percentages).

*BMI* body mass index, *GERD* gastroesophageal reflux disease.

^a^Reported as median (IQR).

When comparing the mean BMI among different levels of asthma control, patients with well-controlled asthma had lower BMI (28.9 ± 7.4 kg/m^2^) compared to the partially controlled group (32.7 ± 6.8 kg/m^2^) and uncontrolled group (32.1 ± 7.4 kg/m^2^) (p = 0.008).

The majority of the patients (66%) suffered from poor sleep quality (PSQI > 5). Almost half of the patients (43%) had a high risk of OSA, 25% had excessive daytime sleepiness (ESS > 10), and 46.5% had significant clinical insomnia (ISI ≥ 10).

### Sleep quality and asthma control levels

**Figure 1:**
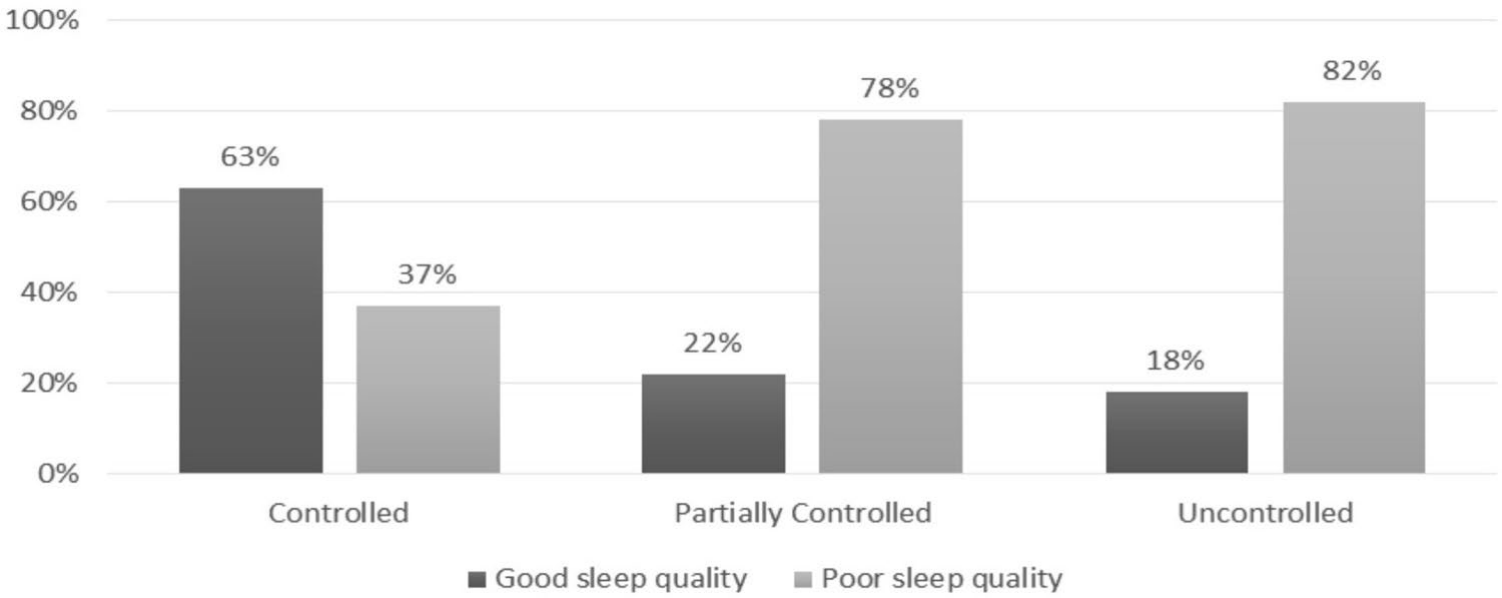
Sleep quality among different level pf asthma control.

There was a strong association between sleep quality and asthma control. Poor sleep quality was less common in patients with well-controlled asthma (37%), compared to those with partially controlled asthma (78%) and uncontrolled asthma (82%) (p ≤ 0.001) (Fig. 1). Furthermore, patients with well-controlled asthma had less excessive daytime sleepiness, a lower risk of OSA, and lower clinical insomnia compared to the partially controlled and uncontrolled asthma patients (p = 0.002, 0.003, and ≤ 0.001, respectively) (Table 2).

**Table 2:** Sleep quality, daytime sleepiness, and insomnia in patients with different levels of asthma control.

	Asthma control levels			p value
	Well-controlled N = 67	Partially controlled N = 55	Uncontrolled N = 78	
PSQI, global score	5.12 ± 3.2	9.07 ± 3.9	9.69 ± 3.9	< 0.001
Good sleep quality, global PSQI ≤ 5	42 (63%)	12 (22%)	14 (18%)	< 0.001
Poor sleep quality, global PSQI > 5	25 (37%)	43 (78%)	64 (82%)	
ESS score	3.69 ± 4.06	6.78 ± 4.2	7.91 ± 4.85	< 0.001
Excessive daytime sleepiness, ESS > 10	7 (10%)	15 (27%)	28 (36%)	0.002
Berlin Questionnaire score	0.94 ± 0.88	1.64 ± 0.95	1.51 ± 1.0	< 0.001
Low risk for OSA	49 (73%)	24 (44%)	41 (53%)	0.003
High risk for OSA	18 (27%)	31 (56%)	37 (47%)	
ISI score	4.96 ± 5.75	10.69 ± 6.73	12.91 ± 6.52	< 0.001
Insomnia, ISI ≥ 10	13 (19%)	27 (49%)	53 (68%)	< 0.001

*PSQI* Pittsburgh sleep quality index, *ESS* Epworth sleepiness scale, *OSA* obstructive sleep apnea, *ISI* insomnia severity index.

### PSQI domains

The mean global PSQI score was lower in the well-controlled group (5.12 ± 3.2) than in the partially controlled (9.07 ± 3.9) and uncontrolled (9.69 ± 3.9) groups (p ≤ 0.001). In addition, the mean score of all PSQI domains was lower in the well-controlled group compared to the partially controlled and uncontrolled groups, with the exception of sleep duration and use of sleep medication, which were only different between well-controlled and uncontrolled groups (Table 3).

**Table 3:** Comparison of the Pittsburgh Sleep Quality Index (PSQI) between patients with different levels of asthma control.

PSQI items	Asthma control levels			p value
	Well-controlled N = 67	Partially controlled N = 55	Uncontrolled N = 78	
PSQI > 5, n (%)	25 (37%)	43 (78%)	64 (82%)	< 0.001
Global PSQI (0–21)	5.12 ± 3.2	9.07 ± 3.9	9.69 ± 3.9	< 0.001
**PSQI domains (0–3)**				
Subjective sleep quality	0.7 ± 0.89	1.29 ± 0.94	1.69 ± 0.68	< 0.001
Sleep latency	0.82 ± 1.0	1.53 ± 0.92	1.64 ± 0.98	< 0.001
Sleep duration	1.06 ± 1.14	1.78 ± 1.23	1.51 ± 1.26	0.004
Sleep efficiency	0.61 ± 0.89	1.45 ± 1.3	1.21 ± 1.25	< 0.001
Sleep disturbances	1.13 ± 0.63	1.53 ± 0.6	1.85 ± 0.58	< 0.001
Use of sleeping medication	0.07 ± 0.4	0.22 ± 0.7	0.38 ± 0.9	0.035
Daytime dysfunction	0.72 ± 0.97	1.27 ± 1.0	1.41 ± 0.97	< 0.001

*PSQI* Pittsburgh Sleep Quality Index.

### Multivariable analysis

Multivariable analysis was performed to identify factors that were strongly associated with poor sleep quality. The following factors were significantly associated with sleep disturbance: high risk for OSA (odds ratio [OR]: 2.46 [95% confidence interval {CI} 1.13, 5.35]), partially controlled asthma (OR: 5.35 [95% CI 2.25, 12.84]), and uncontrolled asthma (OR: 6.63 [95% CI 2.98, 14.75]) (Table 4). No associations between the other covariates and sleep disturbance were found.

**Table 4:** Factors associated with sleep quality in asthma patients.

	Association with poor sleep (PSQI score > 5)				
	Univariate analysis	Multivariable analysis			p-value
	B	p-value	Exp(B)	95% CI for EXP(B)	
Age	0.02	0.07	1.01	(0.99, 1.03)	0.25
BMI	0.05	0.02	0.99	(0.94, 1.04)	0.64
Female gender	0.59	0.06	1.88	(0.88, 4.02)	0.10
+ Allergic rhinitis	0.06	0.85	1.23	(0.62, 2.45)	0.55
+ GERD	− 0.70	0.056	0.47	(0.21, 1.07)	0.07
High risk for OSA	1.19	< 0.001	2.46	(1.13, 5.35)	0.02
**Asthma control**					
Partially controlled	1.80	< 0.001	5.37	(2.25, 12.84)	< 0.001
Uncontrolled	2.04	< 0.001	6.63	(2.98, 14.75)	< 0.001

*BMI* body mass index, *CI* confidence interval, *GERD* gastroesophageal reflux disease, *OSA* obstructive sleep apnea, *PSQI* Pittsburgh Sleep Quality Index.

## Discussion

This study investigated the prevalence of sleep disturbance among Saudi adult asthmatic patients attending pulmonary clinics at KAMC. Approximately two third (66%) of the sample study reported having sleep disturbances. This was similar to results from a previous studies that demonstrated a high prevalence of sleep disturbance among patients with asthma [40–90%]^4–7,31^.The prevalence of uncontrolled asthma was 39%. This was in accordance with a previous studies showing that the prevalence of uncontrolled asthma among asthma patients in pulmonary clinics was 40–82%^7,21,22,32^.

Another important finding of this study was that asthma control had an inverse relationship with sleep disturbance. In comparison to patients with partially controlled or uncontrolled asthma, patients with well-controlled asthma were less likely to report poor sleep quality. Similarly, previous studies from different countries have shown that patients with better asthma control have less sleep disturbance^4,5,14,16,31,33^. Our study also demonstrated sleep disturbances in patients with well-controlled asthma (37%). Braido et al. reported that 10–20% of totally controlled asthma patients still have poor sleep quality^4^. Luyster et al. reported mean PSQI scores > 5 in both severe and non-severe asthma patients^16^.

There was no association between gender or age and sleep disturbance. In contrast, Janson et al. reported that the percentage of asthma patients with difficulties maintaining sleep was higher in females (55%) compared to males (31%). The same study showed that age was associated with a decline in estimated sleep duration^34^.

Asthma patients have a higher prevalence of snoring and OSA compared to the general population, and asthma is an established risk factor for OSA^5,35^. In addition, patients with asthma and OSA are seven times more likely to develop severe asthma^36^. Some factors that might play a role in the interaction between asthma and OSA include local inflammation in the upper airway, allergic rhinitis, systemic inflammation, circulating leptin, neuromechanical reflex bronchoconstriction, intermittent hypoxia, GERD, obesity, and asthma therapy^37^. Julien et al. used overnight home polysomnography to investigate the prevalence and severity of OSA in patients with different levels of asthma. The frequency of OSA-hypopnea events was higher in severe asthmatics compared to moderate asthmatics or non-asthmatics^14^. In the current study, 43% of the asthmatics demonstrated high risk for OSA. Similarly, Lu et al. utilized the STOP-Bang questionnaire to show that 44% of the asthmatics in their cohort were at high risk for OSA^35^. When adjusting for other covariates, our results show that a high risk for OSA was significantly associated with poor sleep quality.

Using multi-regression analysis, the current study shows that having rhino-sinusitis did not increase the chance of having sleep disturbance in asthmatics. This is similar to findings reported in previous studies^4,16^. Nevertheless, Janson et al. reported that allergic rhinitis is associated with difficulty inducing sleep, daytime tiredness, and daytime sleepiness^5^. In our study, patients with co-morbid GERD did not experience increased sleep disturbance compared to those without GERD. Similarly, Luyster et al. reported that the majority of patients with asthma had poor sleep quality regardless of the presence of GERD^16^.

Excessive daytime sleepiness as a result of sleep disturbance has been previously reported among asthma patients^5,7,18,33^. Our study reveals that 27–36% of patients with uncontrolled or partially controlled asthma had excessive daytime sleepiness, whereas only 10% of well-controlled asthma patients had excessive daytime sleepiness. Campos et al. reported significantly increased daytime sleepiness with worse asthma control among 123 women with asthma^33^. Teodorescu et al. also reported a significantly higher percentage of excessive daytime sleepiness in patients with severe asthma (31%), compared to those with non-severe asthma (19%)^18^.

The current study also looked at the relationship between insomnia and asthma control. Insomnia was identified in 68% of patients with uncontrolled asthma, 49% with partially controlled asthma, and 19% with well-controlled asthma. Luyster et al. reported 37% of patients suffering from insomnia among 714 patients with physician-diagnosed asthma, and insomnia was significantly associated with worse asthma control, worse quality of life, and increased depression^17^. Another study showed that the prevalence of insomnia symptoms was common among patients with asthma and asthma-chronic obstructive pulmonary disease overlap (ACO), in comparison to patients without airway diseases^38^.

The current study had several limitations. First, the sample size was small, and it was predominantly female. Second, the study was performed in a single tertiary referral center, which may have introduced referral bias and may not accurately represent the prevalence of the sleep disturbance among asthma patients in the community or in other geographical areas. Third, the cross-sectional design prevented against inferences regarding the directionality of the relationship between sleep quality and asthma control. Lastly, accurate measurements of sleep disturbance, such as sleep studies for OSA and sleep diaries, were not performed.

## Conclusions

Poor sleep quality was common among patients with asthma, especially those with suboptimal levels of asthma control. In addition, patients with uncontrolled asthma had a higher risk for OSA and were more likely to report insomnia and excessive daytime sleepiness. Further studies are needed to better understand the interaction between asthma and sleep.

## Data Availability

The datasets used and/or analysed during the current study are available from the corresponding author on reasonable request.
